# Hyperferritinemia in the elderly can differentiate the bad from the worst: A retrospective cohort analysis

**DOI:** 10.1097/MD.0000000000021419

**Published:** 2020-07-31

**Authors:** Gal Goldhaber, Gad Segal, Amir Dagan

**Affiliations:** a Department of Internal Medicine “T”, Chaim Sheba Medical Center, Affiliated to the Sackler Faculty of Medicine, Tel-Aviv University, Tel-Hashomer; b Rheumatology Unit, Assuta Ashdod Medical Center, Affiliated with Ben-Gurion University of the Negev, Ashdod; c Zabludowicz Center for Autoimmune Diseases, Chaim Sheba Medical Center, Affiliated to the Sackler Faculty of Medicine, Tel-Aviv University, Tel-Hoshomer, Israel.

**Keywords:** elderly, ferritin, hyperferritinemia, in-hospital mortality, prognostication, septic shock

## Abstract

Both total-body iron stores and inflammation influence the concentration of ferritin in the blood. Ferritin as an inflammatory marker might serve as a prognostic marker in the elderly. Therefore, we characterized the clinical circumstances and long-term outcomes of hyperferritinemia (> 1000 μg/L) in hospitalized elderly patients.

A retrospective analysis of elderly (> 70 years) inpatients with ferritin levels of > 1000 μg/L in a tertiary medical center during a 3-year period. We obtained both laboratory and clinical data, assessing the potential association of high ferritin levels with long-term mortality.

Overall, 242 patients (median age 79 years; median ferritin level 1436 μg/L) met the inclusion criteria and were followed for a median time of 18.6 months. Clinical outcomes were dismal for the whole cohort: the diagnosis of solid malignancy occurred in 23.5% of cases while 31% had a severe infection (ranging from sepsis to septic shock). The median survival time of the whole cohort was 4.7 months only. Within the cohort, risk stratification was feasible: higher ferritin levels differentiate between groups of patients who had a poor prognosis (with either septic shock or solid malignancy) and those who had a relatively favorable prognosis (patients diagnosed as suffering from sepsis without shock and patients with iatrogenic causes for hyperferritinemia).

Hyperferritinemia in elderly inpatients is associated with high rates of mortality. Within this group of patients, differential ferritin levels enable further risk stratification. High ferritin levels in the elderly can differentiate the bad from the worst.

## 1 Introduction

Ferritin is a ubiquitous serum protein, known since the beginning of the 20th century. Serving as an iron carrier, and buffer against iron deficiency, measurement of ferritin blood levels is commonly utilized in the evaluation of the total amount of iron in the body.^[1,2]^ Nevertheless, ferritin levels in the blood are influenced not only by the total amount of bodily iron but also by acute and chronic inflammation (serving, most probably, as an iron chelator in face of bacterial invasion) and is thus regarded also as an acute phase reactant.^[3,4]^ Ferritin is also known as a regulator of angiogenesis and has a possible role in the inhibition of cellular immune responses.^[1]^ In daily clinical practice, aside from being a part of anemia investigation, ferritin serves as a diagnostic and prognostic marker of inflammatory diseases such as adult onset Still disease, macrophage activation syndrome, catastrophic antiphospholipid syndrome and septic shock. Moreover, it was suggested to aggregate those conditions under the name “hyperferritinemia syndrome”.^[5,6]^ The non-specific circumstances in which ferritin levels rise make it a possible diagnostic and prognostic marker for other diseases: in a study conducted by Moore et al, in community dwelling adults with ferritin levels above 1000 μg/L it was found that the most common diagnosis was malignancy and the second most common was iron overload syndrome.^[1]^ Others found that an elevated ferritin level was associated with renal failure, hepatocellular injury and even with osteoarthritis.^[7,8]^ Kessler et al showed that in febrile patients undergoing dialysis, high ferritin levels helped to differentiate between patients with bacteremia from those with negative blood cultures.^[9]^ Other studies, which focused on specific patient groups, had partial success in distinguishing infectious and non-infectious etiologies using ferritin levels. For example, patients with fever of unknown origin and higher ferritin levels were more likely to be diagnosed with hematologic disease and inflammatory conditions than with infectious diseases.^[10,11]^ Studies also found that higher ferritin levels could serve as a risk factor for metabolic syndrome^[12]^ and type 2 diabetes mellitus.^[13]^

Ferritin may be a major player in inflammatory pathology through its signaling as part of the innate immune response and through dysregulation of lymphocyte function. However, the precise mechanism by which ferritin contributes to disease in sepsis, rheumatologic, immunologic, and malignant disorders remains elusive. It is suggested that several cytokines, including tumor necrosis factor, interferon-gamma, and numerous interleukins (i.e., interleukin (IL)-1, IL-6, IL-18, IL-33) are implicated in the cytokine cascade leading to extreme Hyperferritinemia.^[14,15]^

The association between high ferritin levels and patients’ mortality is unclear: a number of observational studies conducted in the past could not establish an association between hyperferritinemia^[16–18]^ and increased mortality, while others did show linear correlation between increased ferritin levels and all-cause mortality.^[19,20]^ A possible explanation for the above is the fact that the patients’ populations investigated were heterogeneous. Therefore, we decided to explore the possible prognostic value of hyperferritinemia in the elderly over 70 years. Our study goal was to characterize the morbidities of hyperferritinemia in the elderly and its association with long-term mortality.

## 2 Materials and methods

### 2.1 Study population

After approval of the study protocol by an institutional review board, and waiver acceptance for informed consent, we searched the electronic medical records of the Chaim Sheba Hospital, a tertiary medical center, for all inpatients with serum ferritin levels ≥ 1000 μg/L. All patients over the age of 70 years, hospitalized between January 1, 2012, and December 31, 2015, were included in our study cohort.

### 2.2 Data acquisition and analysis

Many patients had serial ferritin level measurements during their hospital stay. We included in our study only patients who had ferritin > 1000 μg/L on their first, in-hospital measurement. Ferritin levels of over 1000 μg/L were chosen as representatives of extreme hyperferritinemia as was chosen in other studies.^[1]^ We documented other laboratory values, serving as markers for patients’ disease severity (e.g., C - reactive protein, albumin and creatinine blood concentrations). We recorded patients’ diagnoses from discharge letters, and after reviewing all their records, we ascertained the most likely cause of hyperferritinemia, dividing diagnoses into the following groups: metastatic malignancy, sepsis without evidence of shock or septic shock, iatrogenic causes or miscellaneous. Diagnosis of sepsis and septic shock had to meet the criteria of systemic inflammatory response syndrome. The Iatrogenic group of diagnoses included patients who received iron transfusions, blood transfusions, or treated with erythropoietin during the time of or approximating their hospitalization (i.e., patients on chronic dialysis). The group of miscellaneous diagnoses included cases of hepatitis, autoimmune and rheumatologic disorders, as well as unknown causes. When evaluating patient outcomes, we obtained survival data according to the national registry managed by the Israeli Ministry of Health.

### 2.3 Statistical analysis

We described categorical variables using frequency and percentage. We evaluated continuous variables for the normal distribution using histogram and Q-Q plots. We described normally distributed, continuous variables by their mean and standard deviation, while describing non-normally distributed continuous variables by their median and interquartile range. We compared between continuous variables using an independent sample *t* test or a Mann–Whitney *U* test. We evaluated correlation between continuous variables using the Spearman's rank correlation coefficient. Univariate analysis of all-cause mortality throughout the time of follow up was made using Cox regression. We tested the time of follow up with the reverse censoring method. We used Cox regression survival analysis to examine the correlation between the different variables and mortality. Multivariate Cox regression in the backward likelihood ratio method was used to identify independent variables correlated with mortality. Age and gender were included in the first block of the regression using the enter method. Correlation strength was defined as such: correlation of under 0.3 as weak, correlation between 0.3 to 0.6 as colloquial, and a correlation above 0.6 as strong. We added the diagnosis and pathology variables to the regression analysis (Tables 1 and 2). In order to avoid too many predictors in the regression we used backward methods (Wald test was used as criteria for removal) to remove variables with *P*-value > .1 and reported the last step of the regression. All statistical analyses were 1-tailed, and we adjusted *P* values for multiple comparisons using FDR methods, with *P* < .05 being considered as statistically significant. We performed all statistical analysis using IBM SPSS statistics for windows, version 24.0 (IBM Corp. in Armonk, NY).

**Table 1:**
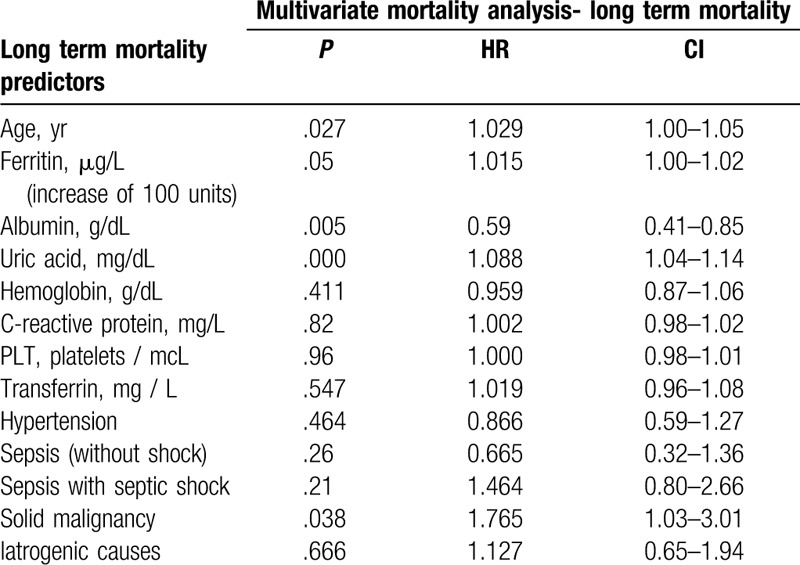
Multivariate analysis. Long term mortality in the cohort before backward method.

**Table 2:**
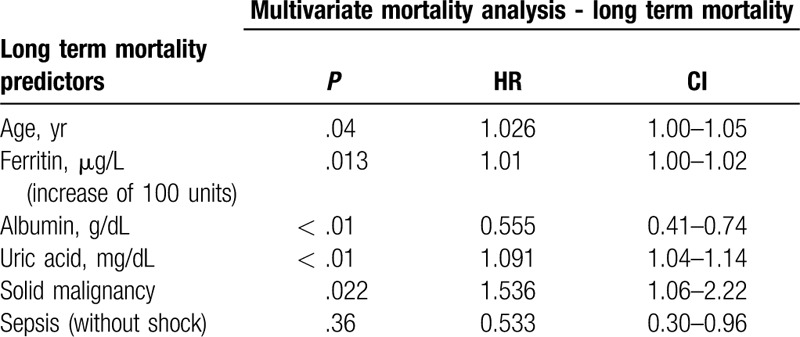
Multivariate analysis. Long term mortality in the cohort after backward method.

## 3 Results

**Figure 1:**
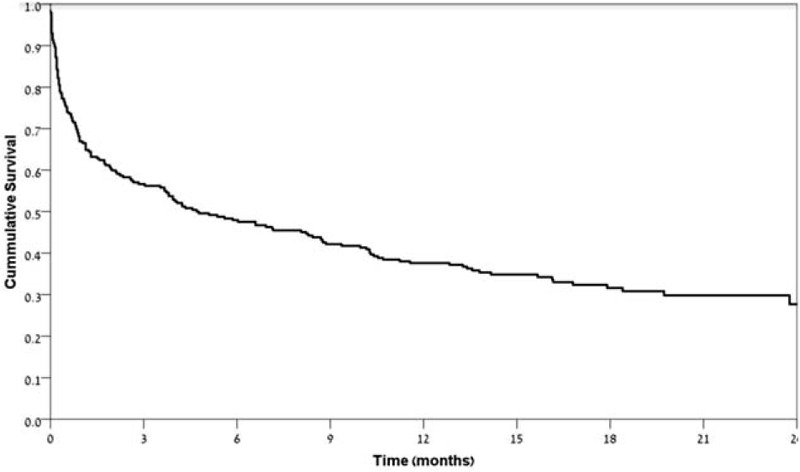
Kaplan–Meier survival curve of the cohort.

Overall 242 patients met the inclusion criteria of our study (age > 70 years and ferritin levels greater than 1000 μg/L). The median time of follow up was 18.6 months. The median age of the cohort was 79 years with an overall male predominance (63.2%). Table 3 describes our patients’ cohort characteristics. The overall prognosis of the patients’ cohort was dismal: nearly 70% (168 patients) of the whole cohort died during the follow-up period (less than 2 years). The median survival time of the cohort was 4.7 months only. Within 6 months only 52.5% of the cohort survived. Mortality rates were even higher when excluding the patients from the iatrogenic groups whose ferritin levels were due to iron transfusions, blood transfusions and EPO. The median survival time of the cohort without the iatrogenic group was only 3.7 months and within 6 months 57.8% of the patients in this group had died (Fig. 1).

**Table 3:**
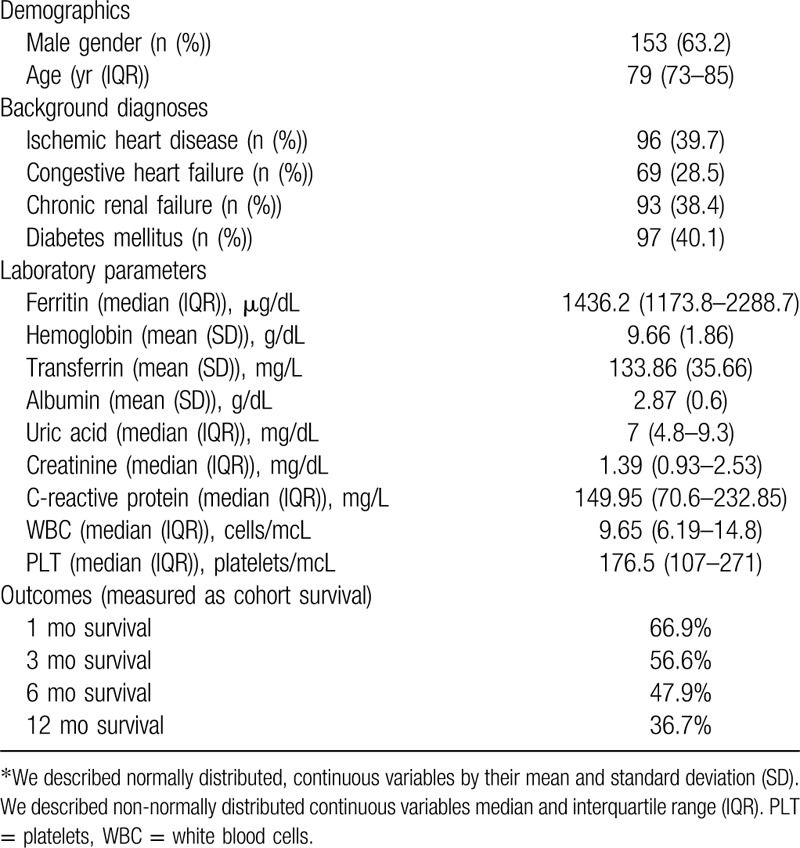
Whole cohort characteristics.

Table 4 details the diagnoses within our cohort that had an association with mortality risk (relative to the whole cohort). Noteworthy, patients with sepsis with no evidence of shock had a much more favorable prognosis: the ferritin levels for septic and non-septic patients were 1181.1 vs 1470.3 μg/L (*P* = .001) carrying a lower risk of mortality (HR = 0.56; *P* < .05). Diagnosis of sepsis with shock, notwithstanding the fact that these patients did not differ significantly in ferritin blood levels from the rest of the cohort (1499 vs 1434 μg/L; *P* = .267), still significantly increased the risk of mortality (HR = 1.48; *P* < .05). Solid malignancy was also common within our study population. Patients with solid malignancy (23.5% of the whole cohort) had a significantly higher ferritin levels compared to patients without solid malignancy (1751.8 vs 1346.9 μg/L; *P* < .001). Higher ferritin levels within this patient population was accompanied by a higher risk of long-term mortality (HR = 1.9; *P* < .01). In contrast, a large patient group within our study group were considered to have iatrogenic causes for hyperferritinemia (e.g. receiving multiple blood and or iron transfusions). These patients, in contrast to the cancer patients, had significantly lower levels of blood ferritin compared to patients who did not have iatrogenic causes for hyperferritinemia (1321.9 vs 1517 μg/L; *P* < .008). The lower levels of ferritin in this group of patients were also associated with a lower risk for 30-days mortality (HR = 0.22; *P* = .001). Existence of chronic comorbidities did not seem to affect ferritin levels within our cohort. Neither gender, arterial hypertension, congestive heart failure, chronic renal failure nor diabetes mellitus carried a significant change in ferritin levels or survival.

**Table 4:**
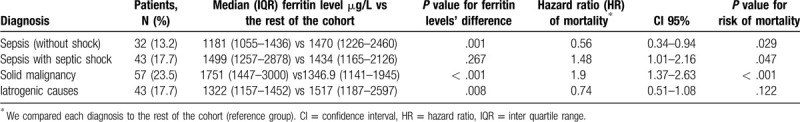
Diagnoses with significant association with blood ferritin levels and risk of mortality.

Both univariate and multi-variate analysis showed that higher ferritin levels, lower albumin levels and higher uric acid levels correlated with short and long-term mortality in a statistically significant pattern. We did not find any correlation with rising C-reactive protein levels. Table 3 includes a multivariate analysis of different laboratory parameters that were thought to influence the mortality of the cohort.

## 4 Discussion

The population worldwide is aging. As part of this phenomenon, we are obliged to find better prognostic factors that will help us improve the treatment of the elderly. In the realm of internal medicine, we aim at exploring the nature of inflammatory, rather than degenerative diseases of the elderly and its influence on mortality. In this study, we sought to characterize the clinical meaningfulness of hyperferritinemia, as a dominant marker of inflammation, amongst elderly, hospitalized patients. This is the first study addressing this issue in hospitalized patients.

We found out, that the overall prognosis of hyperferritinemia, above 1000 μg/L, was dismal: the median survival rate was 4.7 months, with a 6-month mortality rate of 52.5%. This rate was higher when associated with the “non-iatrogenic” group of diagnoses. Counterintuitively, the classic hyperferritinemia entities, for example adult onset Still disease or hemophagocytic syndrome were missing from our cohort of elderly patients, replaced in turn by metastatic malignancies and septic shock.

We found that high levels of ferritin were associated with increased mortality even after correction to potential confounders including C-reactive protein, albumin, platelet count, transferrin, hemoglobin, uric acid concentrations, and white blood cell counts. Moreover, other inflammatory markers used for prognostication in daily practice, such as C- reactive protein, platelets, and white blood cells were found not to be independently associated with mortality in this cohort.

When trying to characterize this population and its different pathologies, we found that a significant percentage of patients were diagnosed with septic shock, sepsis and metastatic malignancy which was in keeping with the findings of Moore et al.^[1]^ As expected, these diagnoses were correlated with higher rates of short and long-term mortality in this population.

The association between hyperferritinemia and specific pathologies was demonstrated in several recent studies. In contrast to the pediatric population, rheumatologic diseases are only rarely associated with extreme hyperferritinemia in elderly.^[1,7]^ Within our cohort of elderly population, we found similar pathologies to those observed in other recent studies: 28.5% of our cohort had metastatic solid malignancy, 9.5% had hematologic malignancy and 31% had infection, ranging in severity from sepsis to septic shock. Less than 5% of our cohort had rheumatologically or chronic systemic inflammatory diseases as their presumed etiology for hyperferritinemia.

The association between mortality rates and hyperferritinemia has been researched in a growing number of studies in the past years.^[20–23]^ The poor prognosis in patients with hyperferritinemia attributed to malignancy was already reported in a previous study,^[24]^ but we suggest that this finding can be generalized to other disease states as well. According to our study, it seems that when patients over the age of 70 years are admitted to the hospital, for whatever reason, with ferritin levels above 1000 μg/L, their chances of surviving the next 6 months are less than 50%.

### 4.1 Study limitations

We designed this study as a retrospective, observational study. Therefore, causality of associations cannot be inferred. In addition, we did not have an external control group. All associations and comparisons were made within the group of elderly patients with hyperferritinemia. Further, prospective, interventional studies are needed in order to establish our findings and look for therapeutic options that would both better the elderly patients’ prognosis alongside a measurable decline in ferritin levels.

## 5 Conclusions

In conclusion, our study demonstrated that elderly inpatients with high ferritin have a high mortality rate. Those patients will usually suffer from malignancy or sepsis and not from chronic benign inflammatory diseases. These findings are of the utmost importance in the hospitalized population, as they may help the clinician in more rapid diagnosis and in determining prognosis and in so doing aiding in decision making. High ferritin levels in the elderly can differentiate the bad from the worst.

## Author contributions

Gal Goldhaber (study design; data collection; statistical analysis; manuscript writing and proof). Gad Segal (study design; data collection; statistical analysis; manuscript writing and proof). Amir Dagan (study design; data collection; statistical analysis; manuscript writing and proof).
